# The Notch inhibitor cowanin accelerates nicastrin degradation

**DOI:** 10.1038/s41598-018-23698-4

**Published:** 2018-03-29

**Authors:** Midori A. Arai, Ryuta Akamine, Anna Tsuchiya, Tatsuro Yoneyama, Takashi Koyano, Thaworn Kowithayakorn, Masami Ishibashi

**Affiliations:** 1 Graduate School of Pharmaceutical Sciences, Chiba University, 1-8-1 Inohana, Chuo-ku, Chiba, 260-8675 Japan; 2 Temko Corporation, Tokyo, Japan; 3 Faculty of Agriculture, Khon Kaen University, Khon Kaen, Thailand

## Abstract

Aberrant activation of Notch signaling contributes to the pathogenesis of several different types of cancer, and Notch pathway inhibitors may have significant therapeutic potential. Using a unique cell-based assay system, we isolated twelve compounds, including one new natural product from *Garcinia speciosa*, that inhibit the Notch signaling pathway. *HES1* and *HES5* are target genes of the Notch cascade, and compound **2**, referred to as cowanin, decreased the protein levels of HES1 and HES5 in assay cells. Furthermore, cowanin (**2**) showed potent cytotoxicity against human leukemic HPB-ALL cells. The Notch signaling inhibitory activity of cowanin (**2**) is linked to the increased degradation of nicastrin, which is one of the components of the γ-secretase complex. To the best of our knowledge, this is the first example of a compound with Notch pathway inhibitory activity mediated by nicastrin degradation.

## Introduction

Notch signaling plays a pivotal role in the regulation of many fundamental cellular processes, including proliferation and stem cell maintenance. It also influences cell differentiation decisions at various stages during embryonic and adult development^1,2^. It is apparent that an important direct output of Notch activity is the upregulation of basic helix-loop-helix (bHLH) transcriptional repressors such as the *hairy and enhancer of split 1* (*HES1*), *HES5* and *HES* related (*HESR/HEY*) family genes. Notch signaling has an important role in the regulation of neural stem cell differentiation^3,4^, and the differentiation of neural stem cells into neurons can be inhibited by Notch signaling. In direct contrast, the Notch pathway also promotes astrocyte differentiation from glial progenitors derived from neural stem cells. Aberrant upregulation of Notch signaling is oncogenic in multiple hematologic and solid malignancies^5–7^. For example, activating mutations of *NOTCH1*, a transmembrane receptor that regulates normal T cell development, drives the development of human T-cell acute lymphoblastic leukemia (T-ALL)^8^. Notch has been proven to be an oncoprotein in melanocytes^9^, prostate^10^, and breast^11^ cancer cells, providing significant impetus for the development of clinically effective Notch pathway inhibitors^12^.

**Figure 1:**
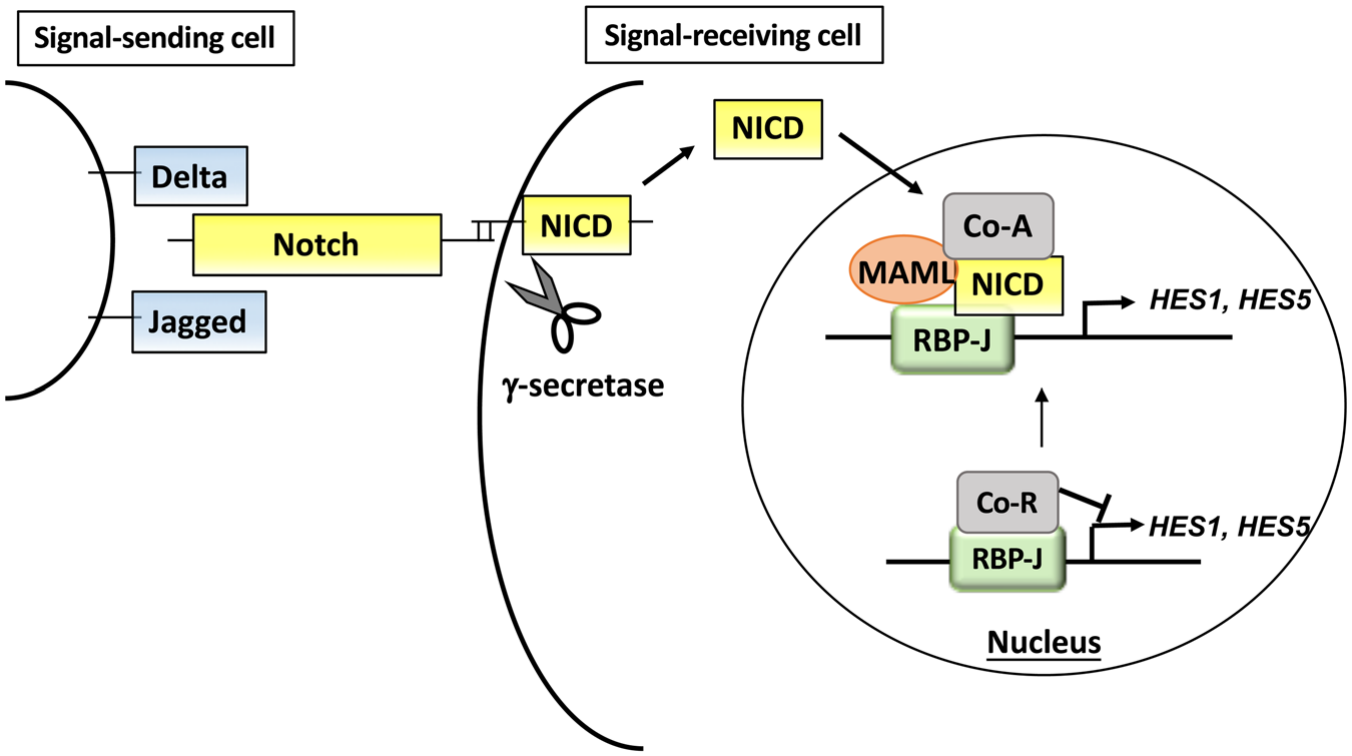
The Notch signaling pathway. Interaction between Notch and Delta or Jagged that exist on the surface of signal-sending cells triggers the cleavage of Notch protein by ADAM/TACE metalloproteases and γ-secretase to produce NICD. NICD moves to the nucleus to forms a heterotrimer with RBP-J and MAML to enhance transcription of target genes, such as *HES1 and HES5*. NICD: Notch intracellular domain, MAML: mastermind-like protein (co-activator), HES1: hairy and enhancer of split 1, HES5: hairy and enhancer of split 5.

Signal transduction from Notch receptors is shown in Fig. 1. Notch signaling is activated by interaction between the ligand-expressing cell and the signal-receiving cell. The binding of Jagged or Delta ligand proteins to the Notch receptor induces a conformational change, exposing the S2 cleavage site in the Notch receptor to the metalloproteinase containing protein, ADAM/TACE. Following S2 cleavage, the subsequent S3 cleavage is mediated by γ-secretase, which is a multi-subunit complex composed of presenilin 1 (PS1), nicastrin, presenilin enhancer 2 (PEN-2) and anterior pharynx-defective 1 (APH-1). The S3 cleavage results in the release of the Notch intracellular domain (NICD), which translocates to the nucleus and forms a heterotrimer with mastermind-like protein (MAML) and DNA binding protein recombination signal binding protein for immunoglobulin kappa J region (RBP-J) to initiate transcription of target genes.

Various Notch inhibitors have been reported including γ-secretase inhibitors, DAPT (*N*-[*N*-(3,5-difluorophenacetyl)-L-alanyl]-*S*-phenylglycine *t*-butyl ester)^13^, RO4929097^14^, MK-0752^15^ and PF0384014^16^. SHAM1, a MAML1-derived peptide that inhibits the binding of full-length MAML1 to NICD1-RBP-J, was also reported as a potent Notch signaling inhibitor^17^. The natural products, curcumin^18^ and genistein^19^, downregulate Notch1. Butein was also reported as a potent inhibitor^20^. A peptide-based protein knockdown system was also developed for degradation of Notch1^21^. Efforts have been taken to develop Notch signaling inhibitors as agents with clinically used chemotherapeutics to treat cancer^12^. However, the development of Notch inhibitors from natural products has been limited despite the increasing realization that natural products are a valuable repository of potentially effective new cancer drugs^22^. In this study, we searched for new Notch signaling inhibitors from natural resources using a unique cell-based reporter assay recently developed by our group.

## Results and Discussion

**Figure 2:**
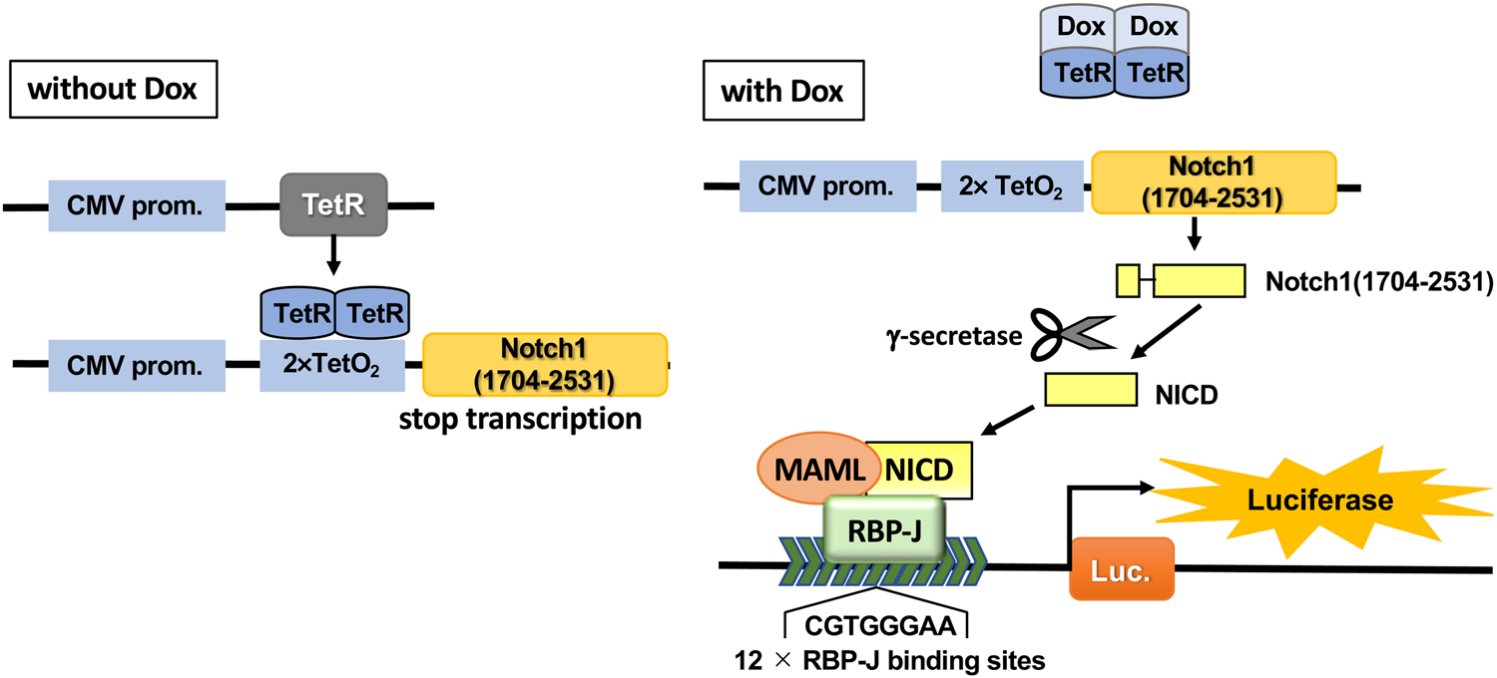
The T-REx assay system. Without Dox, expression of Notch1(1704–2531) is suppressed by TetR. The addition of Dox inactivates TetR to initiate expression of Notch1(1704–2531), which is then cleaved by endogenous γ-secretase to form NICD. NICD then translocates to the nucleus and forms a heterotrimer with RBP-J and MAML that binds to a 12× RBP-J binding site to drive luciferase expression. Luciferase activity was detected as Notch signaling pathway activity. Dox: doxycycline, TetR: tetracycline repressor, Luc.: luciferase.

**Figure 3:**
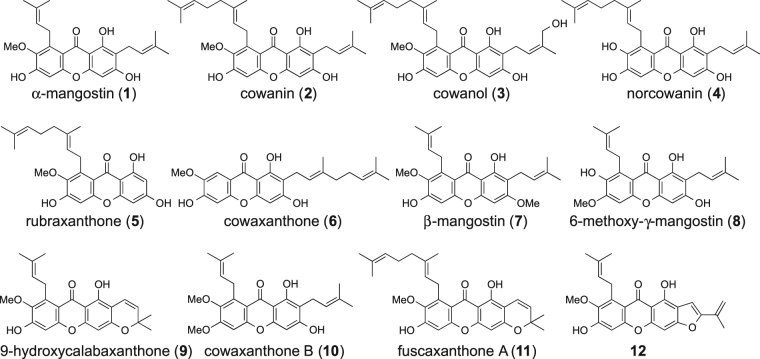
The structures of isolated compounds. Compound **12** was isolated as a new natural product.

We constructed a cell-based reporter assay with the T-Rex system to evaluate Notch signaling inhibitory activity^23^. Without doxycycline (Dox), the tetracycline repressor (TetR) inhibits the expression of N-terminally FLAG-tagged mouse Notch1(1704–2531), which is 40 aa longer than NICD at the N-terminus. In the presence of Dox, Dox binds to the TetR to release the inhibition of expression of Notch1(1704–2531). Cleavage of Notch1(1704–2531) by γ-secretase generates NICD, which then translocates to the nucleus and forms a heterotrimer with RBP-J driving luciferase transcription (Fig. 2). This assay cells (LS174T Notch cells) was stably incorporated with pGL4.20 luciferase plasmid which has 12 × RBP-J binding site (12 copies of CGTGGGAA)-β-globin promoter. Cell viability was measured using a fluorometric microculture cytotoxicity assay^24^. Screening of our plant extract library showed that a MeOH extract of *Garcinia speciosa* includes Notch inhibitors in the crude extract. A 100 μg/mL MeOH extract reduced luciferase activity to 44% with cell viability of 79%. The composition of the *G. speciosa* MeOH extract was partitioned successively with hexane, EtOAc, *n*BuOH, and water. The combined hexane and EtOAc layer was investigated using chromatographic techniques (Diaion HP-20, silica gel and ODS), resulting in the isolation of α-mangostin (**1**)^25^, cowanin (**2**)^25^, cowanol (**3**)^25^, norcowanin (**4**)^26^, rubraxanthone (**5**)^27^, cowaxanthone (**6**)^28^, β-mangostin (**7**)^28^, 6-methoxy-γ-mangostin (**8**)^29^, 9-hydroxycalabaxanthone (**9**)^25^, cowaxanthone B (**10**)^30^, fuscaxanthone A (**11**)^31^ and a new compound (**12**) (Fig. 3).

**Figure 4:**
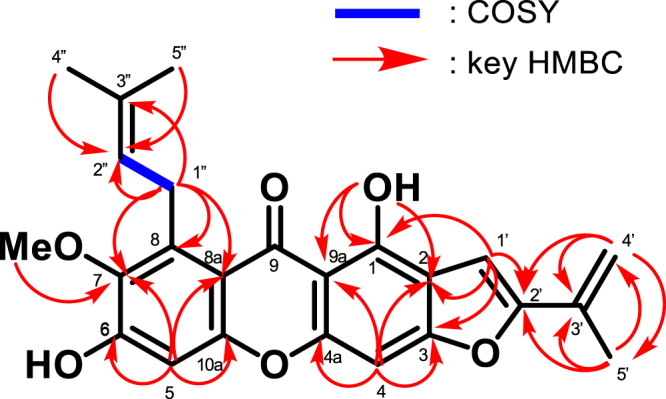
Key ^1^H-^1^H COSY and HMBC data for **12**.

Compound **12** was isolated as a yellow solid. The HR-ESI-MS showed a pseudo molecular ion peak [M-H]^−^ at *m/z* 405.1327 (calcd for C_24_H_21_O_6_, ∆ -1.1 mmu), which indicated the molecular formula, C_24_H_22_O_6_. The IR absorption at 3371 and 1607 cm^−1^ indicated the presence of an O-H group and C=O group, respectively. From the ^1^H-NMR spectrum, two characteristic signals of the xanthone were observed at δ_H_ 6.87 (s, 1H, H-4) and δ_H_ 6.85 (s, 1H, H-5) and a chelated hydroxyl proton at δ_H_ 14.10 (s, 1H, 1-OH). The existence of a prenyl side chain was deduced from the signals at δ_H_ 4.09 (d, 2H, *J* = 5.8 Hz, H-1′′), 5.28 (1H, m, H-2′′), 1.83 (s, 3H, H-4′′) and 1.69 (s, 3H, H-5′′). From the similarity to mangaxanthone A^32^, compound **12** was presumed to have the furan-2-yl moiety with a 1-methylethenyl portion. The ^13^C-NMR spectrum revealed 24 carbons. The position of the prenyl side chain at C-8 was confirmed by the HMBC correlation between the methylene proton at δ_H_ 4.09 (d, 2H, *J* = 5.8 Hz, H-1′′) with C-8 (δ_C_ 137.1), C-7 (δ_C_ 142.5) and C-8a (δ_C_ 111.7), while the position of the methoxy group at C-7 was determined by the correlation of the methoxy proton at δ_H_ 3.79 (s, 3H) with C-7 (δ_C_ 142.5) (Fig. 4). The position of a 1-methylethenyl group was determined by the HMBC correlations between methyl proton at δ_H_ 1.69 (s, 3H, H-5′) and C-2′.

**Figure 5:**
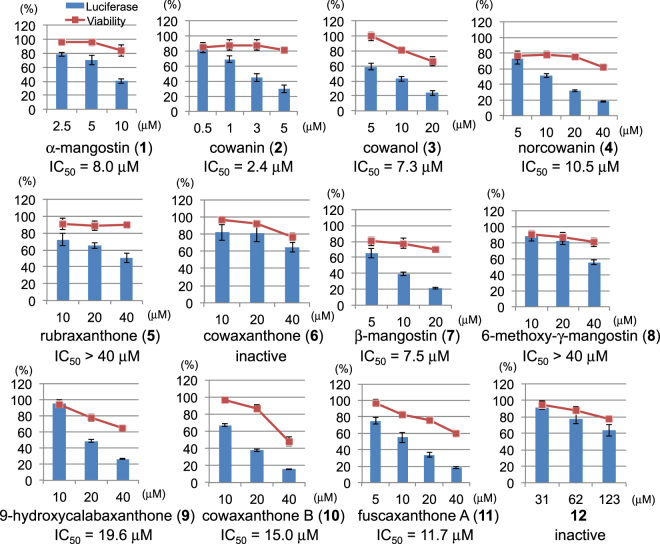
Inhibition of Notch transcriptional activity and cell viability by **1**–**12**. Assay cells (LS174T Notch cells: 2 × 10^4^ cells/well) were incubated in a 96-well white plate at 37 °C for 12 h. After incubation, 50 ng/mL Dox was added to each well to induce expression of exogenous Notch1(1704–2531) protein. After incubation at 37 °C for 12 h, the medium was changed to Dox-free medium containing individual samples. Luciferase activity was measured after treatment at 37 °C for 12 h. Cell viability was evaluated by the FMCA method at the same time. These assays were performed in 0.1% DMSO (n = 3). Error bars represent SD.

Next, we investigated the Notch signal inhibitory activity of the twelve compounds (Fig. 5). Compounds **1**, **2**, **3** and **7** showed potent inhibitory activity with IC_50_ values of less than 10 μM (8.0, 2.4, 7.3 and 7.5 μM, respectively). Compounds **4**, **9**, **10** and **11** had moderate activity with IC_50_ values of 10.5, 19.6, 15.0 and 11.7 μM, respectively. The remaining compounds, **5**, **6**, **8** and **12**, had weak activity or were inactive. The Structure-Activity Relationship (SAR) of isolated Notch inhibitors indicated that two prenyl moieties at C-2 and C-8 strengthened activity. From the comparison of the activities of compounds **1** and **10**, the OH group at the C-6 position was preferred over the methoxy group. The methoxy group at the C-7 position seemed to be preferred for activity. In addition, the ring portions at C-2 and C-3 weakened activity based on comparisons of **1** with **9** or **12**, and **2** with **11**.

**Figure 6:**
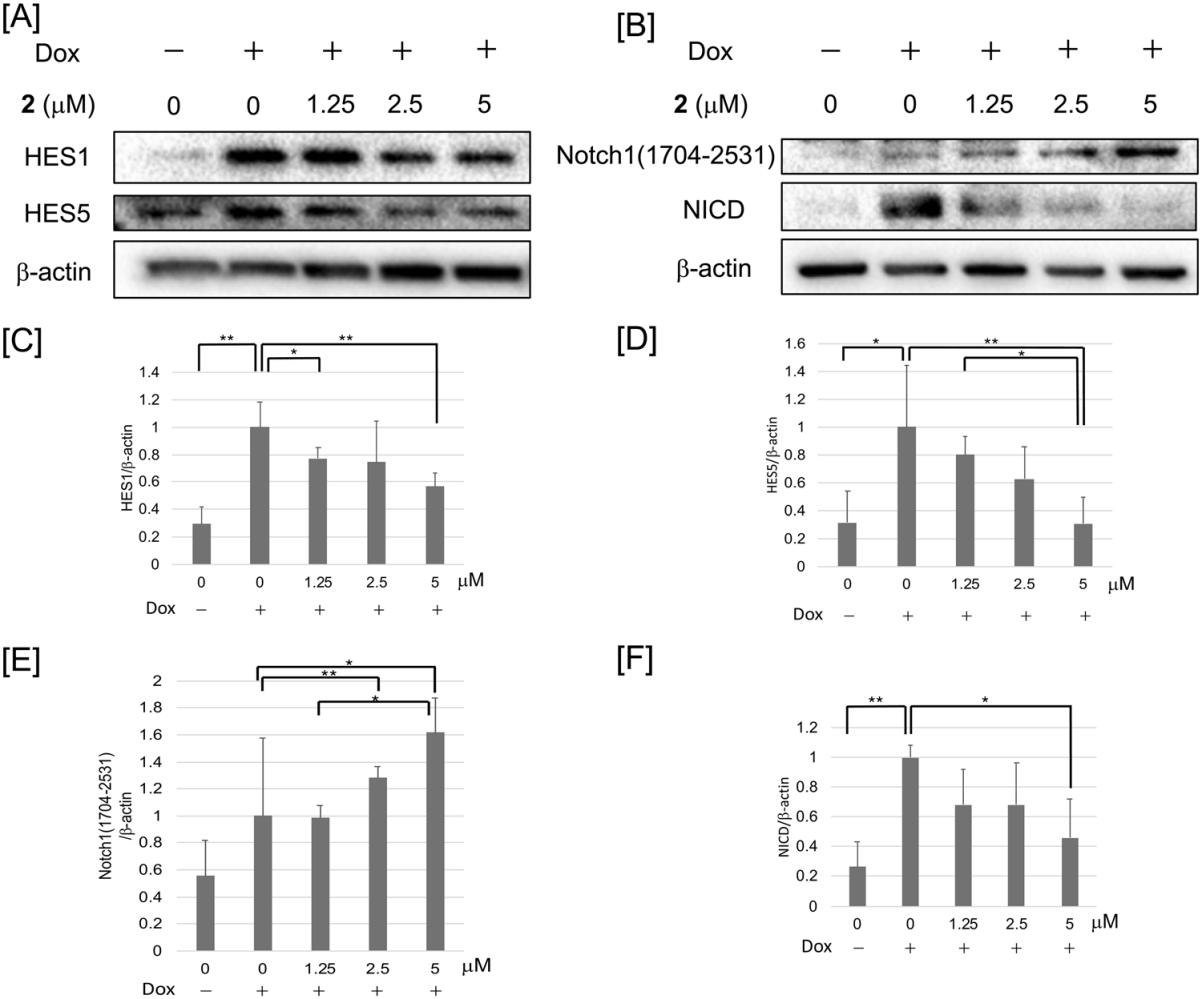
Inhibition of protein expression by cowanin (**2**) in assay cells. Assay cells (LS174T Notch cells: 1 × 10^6^ cells/dish) were incubated in a 6-cm dish for 12 h at 37 °C. After another 12 h incubation with 50 ng/mL Dox, and cells were washed with PBS. Medium was changed to fresh medium with **2** and incubated for 12 h. The effect on protein expression was analyzed by Western blotting. β-Actin was used as internal control. (**A**) Western blot analysis of HES1 and HES5 expression in assay cells after treatment with cowanin (**2**). (**B**) Western blot analysis of Notch1(1704–2531) and NICD expression in assay cells after treatment with cowanin (**2**). (**C**–**F**) Relative protein levels analyzed by densitometry. Data are presented as the mean with SD from three independent experiments. *P < 0.05 and **P < 0.01 are considered statistically significant. *P* values were analyzed by Student’s *t* test.

We focused on compound **2**, referred to as cowanin, which had the strongest inhibitory activity. The expression of the Notch signaling target proteins, HES1 and HES5, was reduced by the addition of cowanin (**2**) to the assay cells (Fig. 6A,C,D). NICD was reduced dose dependently (Fig. 6B,F), while the expression of Notch1(1704–2531) was increased (Fig. 6B,E). This result indicated that cowanin (**2**) might affect the activity of the γ-secretase complex.

**Figure 7:**
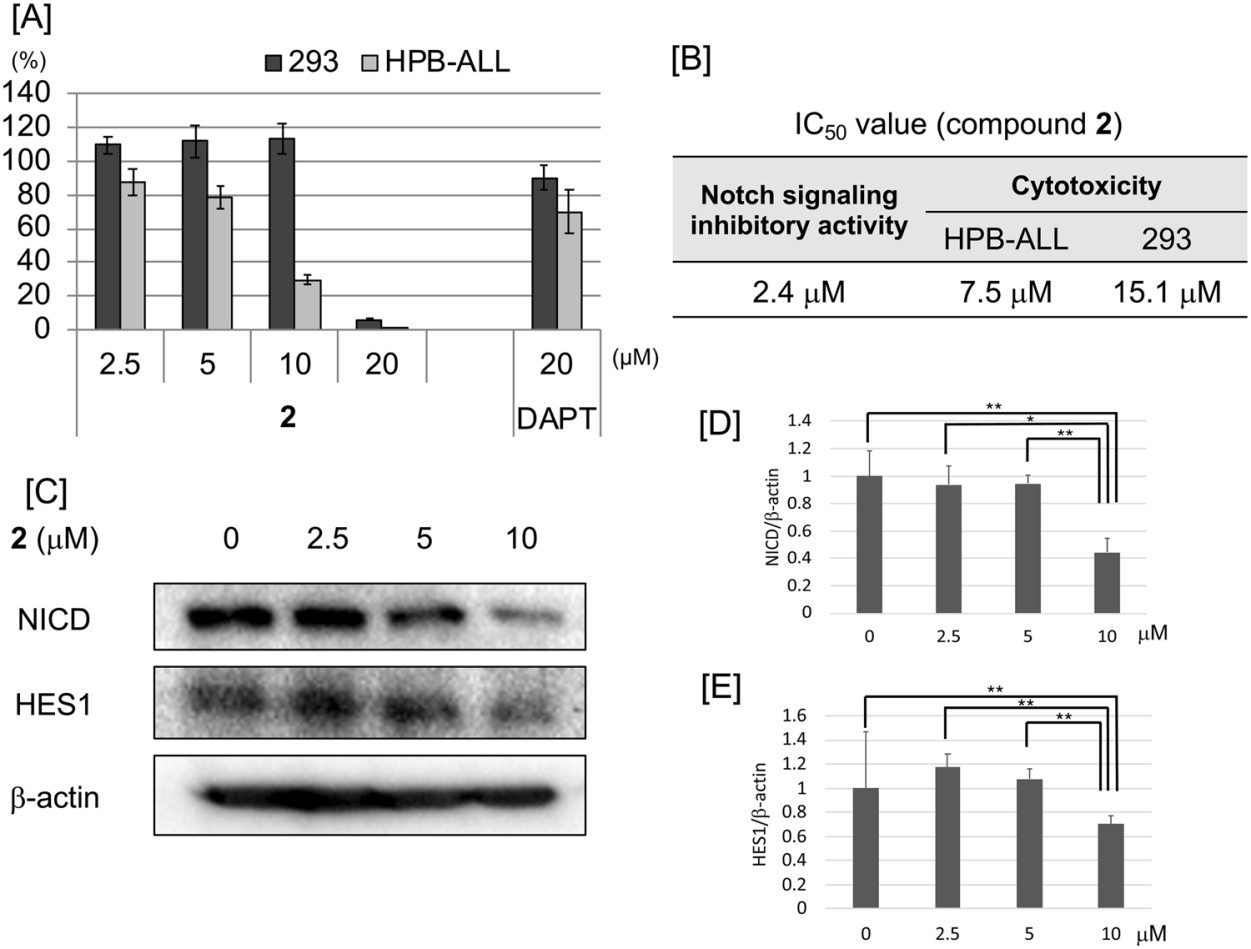
Effect of cowanin (**2**) on HPB-ALL cells. (**A**) Cytotoxicity of **2** and DAPT against 293 and HPB-ALL cells. HPB-ALL cells (1 × 10^4^ cells per well) were incubated in 96-well black plates at 37 °C for 72 h. Media also contained different concentrations of compound. After incubation, AlamarBlue was added 10 μL per well. After incubation for 4 h, using a fluorescence plate reader, fluorescence was measured and cell viability was determined. Data are presented as the mean with SD from three independent experiments. (**B**) IC_50_ values of cowanin (**2**) in Notch signaling inhibitory activity and cytotoxicity. (**C**) Western blot analysis of NICD and HES1 expression in HPB-ALL cells after treatment with **2**. HPB-ALL cells (1 × 10^6^ cells/dish) were incubated in a 6-cm dish for 72 h at 37 °C with **2**. The effect on protein expression was analyzed by Western blotting. β-Actin was used as internal control. (**D**,**E**) Relative protein levels of NICD and HES1 analyzed by densitometry. Data are presented as the mean with SD from three independent experiments. *P < 0.05 is considered statistically significant. *P* values were analyzed by Student’s *t* test.

To investigate the effect of cowanin (**2**) on cancer cells, the cytotoxicity of the compound on the T-ALL cell line, HPB-ALL, was evaluated (Fig. 7A). Cowanin (**2**) reduced the viability of HPB-ALL cells at 10 μM, while the viability of normal human embryonic kidney 293 cells was not affected, although cytotoxicity was detected at 20 μM. Cowanin (**2**) showed stronger cytotoxicity than that of the known γ-secretase inhibitor DAPT (Fig. 7A). Although IC_50_ of Notch signal inhibitory activity of DAPT in our luciferase assay system was 0.12 μM, cowanin (**2**) showed high efficient cytotoxicity against cancer cells HPB-ALL than that of DAPT (IC_50_ >20 μM) (Fig. 7A). The IC_50_ values of cowanin (**2**) were 7.5 μM (HPB-ALL cells) and 15.1 μM (293 cells) (Fig. 7B). This difference of cytotoxicity between cancer cells and normal cells is also attractive. The expression of NICD and HES1 was reduced in HPB-ALL cells at 10 μM significantly (Fig. 7C–E).

**Figure 8:**
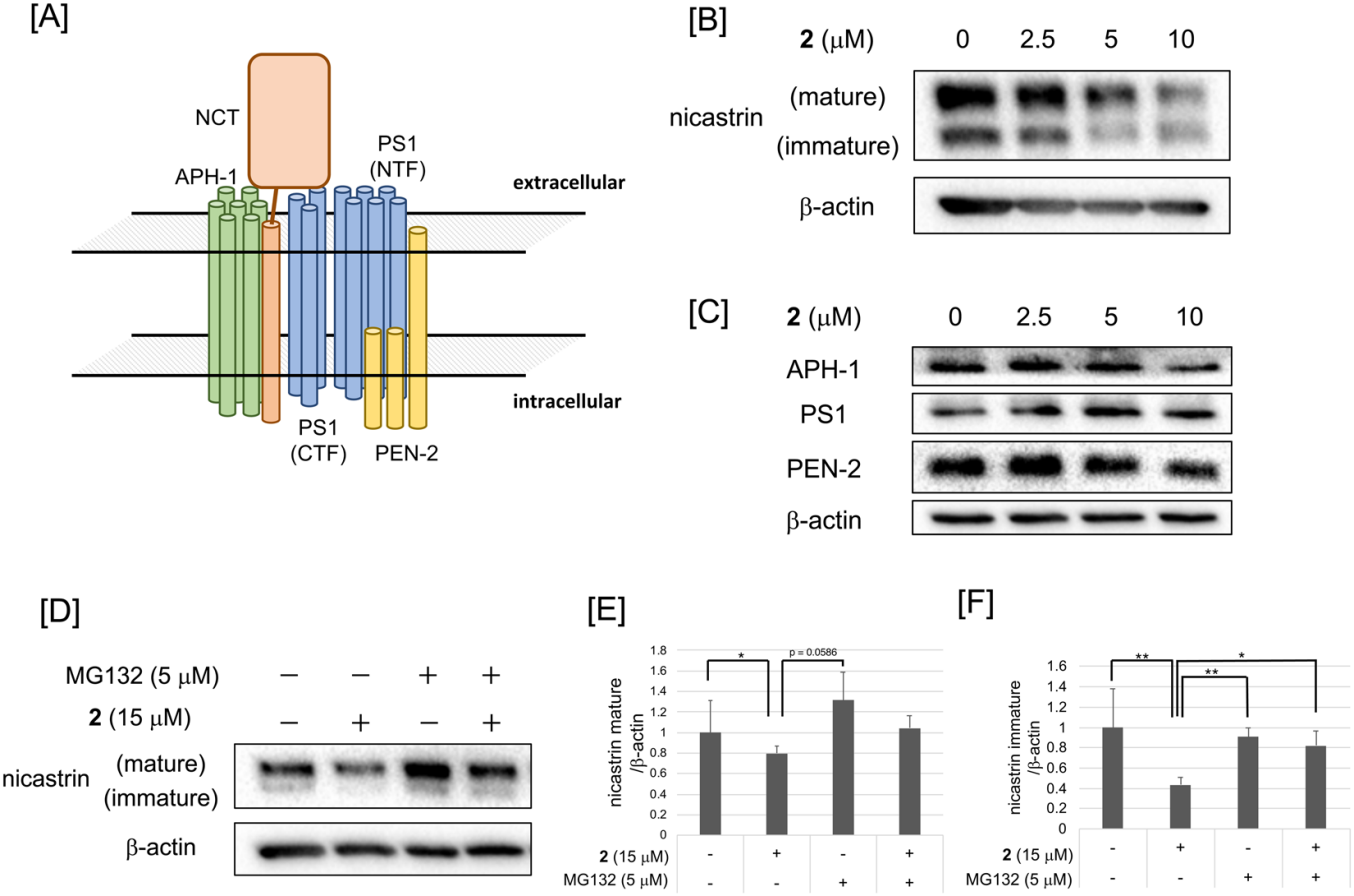
Inhibition of expression of components of the γ-secretase complex by cowanin (**2**) in HPB-ALL cells. (**A**) Schematic representation of γ-secretase complex. γ-Secretase is composed of four membrane proteins: presenilin1 (PS1), nicastrin (NCT), anterior pharynx defective 1 (APH-1) and presenilin enhancer 2 (PEN-2). (**B**,**C**) Western blot analysis of nicastrin, APH-1, PS1 and PEN-2 expression in HPB-ALL cells after treatment with cowanin (**2**). HPB-ALL cells (1 × 10^6^ cells/dish) were incubated in a 6-cm dish for 72 h at 37 °C with **2**. The effect on protein expression was analyzed by Western blotting. β-Actin was used as internal control. (**D**) MG132 abrogated the reduction of nicastrin expression by cowanin (**2**). HPB-ALL cells (1 × 10^6^ cells/dish) were incubated in a 6-cm dish for 12 h at 37 °C with **2**. The effect on protein expression was analyzed by Western blotting. β-Actin was used as internal control. (**E**,**F**) Relative protein levels of nicastrin mature and immature analyzed by densitometry. Data are presented as the mean with SD from three independent experiments. *P < 0.05 and **P < 0.01 are considered statistically significant. *P* values were analyzed by Student’s *t* test.

The effects of cowanin (**2**) on the expression of components of the γ-secretase complex (Fig. 8A), nicastrin (Fig. 8B), APH-1, PS1 and PEN-2 (Fig. 8C) were evaluated to address the mechanisms by which cowanin (**2**) inhibits γ-secretase activity. It was known that the activity site exists in PS1 but the complexation of these four proteins is important for activity^33^. Although cowanin (**2**) didn’t affect the expression of APH-1, PS1 and PEN-2, nicastrin levels were reduced (Fig. 8B). To address the possibility that cowanin (**2**) accelerates the degradation of nicastrin, a proteasome inhibitor, MG132, was co-incubated with cowanin (**2**) (Fig. 8D–F). We confirmed that the cell viability was 98% under 12 h treatment of cowanin (**2**) (15 μM). When MG132 (5 μM) was added to HPB-ALL cells with cowanin (**2**) (15 μM), the decrease in nicastrin expression was not so marked. These data indicated that the mechanism of Notch inhibition by cowanin (**2**) was acceleration of nicastrin degradation. As Fig. 8F showed, cowanin (**2**) decreased immature nicastrin stronger than mature nicastrin. Nicastrin was known to be matured by glycosylation of a large extracellular domain. And PS1 interacts preferentially with mature nicastrin^34^. Therefore, hydrophobic compound, cowanin (**2**), might prefer to bind immature extracellular domain without glycans. By cowanin (**2**) binding, PS1 binding to nicastrin might be reduced. That might cause to increase the amount of unstable free nicastrin, then degradation of nicastrin might be accelerated.

## Conclusion

In conclusion, we report the isolation of twelve compounds, including one new natural product, using our cell-based assay for Notch signal inhibitors. Cowanin (**2**) was shown to inhibit γ-secretase activity by reducing nicastrin levels, which was the result of the acceleration of nicastrin degradation. To the best of our knowledge, this is the first example of a compound that accelerates nicastrin degradation and shows Notch inhibitory activity.

## Methods

### General Experimental Procedures

Optical rotations were recorded on a JASCO P-1020 polarimeter. NMR spectra were recorded on JEOL ECP600 and ECZ600 spectrometers in a deuterated solvent whose chemical shift was taken as an internal standard. ESI-MS were obtained on a JEOL JMS-T100LP. Silica gel 60 N (Kanto Chemical Co., Inc., Tokyo, Japan), Chromatorex ODS and Chromatorex PSQ100B (Fuji Silysia Chemical Ltd., Kasugai, Japan) were used for column chromatography. Luna Phenyl-Hexyl (φ 10 × 250 mm) (Phenomenex Torrance, CA) was used for Preparative HPLC. Nano Drop 2000 spectrophotometer (Thermo Fisher Scientific Inc., Waltham, USA) was used for protein concentrations measurement.

### Plant materials

*Garcinia speciosa* (Clusiaceae) was collected from Thailand in 2013, and identified by one of the authors (T. Kowithayakorn). A voucher specimen (KKP415) was used for deposit at the Department of Natural Products Chemistry, Graduate School of Pharmaceutical Sciences, Chiba University.

### Extraction and isolation of compounds

The MeOH extract (21.8 g) of *G. speciosa* (bark; 86 g) was suspended in 10% aq. MeOH (250 mL) and partitioned between hexane, EtOAc, and BuOH (250 mL × 3) to give hexane (6.4 mg), EtOAc (17.7 g), BuOH (4.5 g), and aqueous (2.0 g) soluble layers. Chlorophyll was removed from a combined mixture of active hexane and EtOAc soluble layers by Diaion HP-20 (φ 80 × 280 mm) with the MeOH:acetone (1:0–0:1). An active fr. 1B (3.5 g) was separated by silicagel column chromatography (φ 40 × 220 mm; CHCl_3_-MeOH = 50:1–0:1, and 0.1% TFA in MeOH) to give the fractions 2A to 2J. By ODS column chromatography (φ 15 × 210 mm; H_2_O-MeOH = 15:85–0:1, and 0.1% TFA in MeOH), fr. 2A (72.4 mg) was separated to the fractions 7A to 7G. Fr. 7B (4.1 mg) was subjected to HPLC (Luna Phenyl-Hexyl; MeOH-H_2_O = 85:15, flow rate 2.0 mL/min) to afford compound **8** (1.4 mg, t_*R*_ 31.6 min) and compound **1** (0.7 mg, t_*R*_ 36.8 min). Fr. 7D (31.2 mg) was subjected to HPLC (Luna Phenyl-Hexyl; MeOH-H_2_O = 90:10, flow rate 2.0 mL/min) to give the fractions 9A to 9F and gave compound **2** (0.5 mg, t_*R*_ 31.6 min) and compound **7** (2.4 mg, t_*R*_ 34.0 min). Fr. 9B was further separated by HPLC (Luna Phenyl-Hexyl; MeOH-H_2_O = 83:17, flow rate 2.0 mL/min) to afford compound **9** (1.2 mg, t_*R*_ 99.0 min) and compound **10** (1.6 mg, t_*R*_ 107.0 min). Fr. 2B (1634.5 mg) was separated by ODS column chromatography (φ 30 × 300 mm; H_2_O-MeOH = 15:85–0:1, and 0.1% TFA in MeOH) to give the fractions 3A to 3G. Fr. 3C (49.8 mg) was subjected to HPLC (Luna Phenyl-Hexyl; MeOH-H_2_O = 87:13, flow rate 2.0 mL/min) to afford compound **5** (1.2 mg, t_*R*_ 24.2 min), compound **6** (1.8 mg, t_*R*_ 25.0 min), compound **1** (22.0 mg, t_*R*_ 29.4 min) and compound **4** (0.5 mg, t_*R*_ 36.0 min). Fr. 3E (55.6 mg) was subjected to HPLC (Luna Phenyl-Hexyl; MeOH-H_2_O = 90:10, flow rate 2.0 mL/min) to afford compound **1** (2.3 mg, t_*R*_ 17.5 min) and compound **2** (21.8 mg, t_*R*_ 27.2 min). Fr. 2 C (1119.0 mg) was separated by ODS column chromatography (φ 30 × 290 mm; H_2_O-MeOH = 15:85–0:1, and 0.1% TFA in MeOH) to give the fractions 4A to 4H. Fr. 4E (26.7 mg) was subjected to HPLC (Luna Phenyl-Hexyl; MeOH-H_2_O = 85:15, flow rate 2.0 mL/min) to give compound **3** (15.6 mg, t_*R*_ 30.0 min) and compound **4** (0.6 mg, t_*R*_ 42.8 min). An active fr. 1C was separated by silicagel column chromatography (φ 32 × 250 mm; CHCl_3_-MeOH = 1:0–0:1, and 0.1% TFA in MeOH) to afford the fractions 11A to 11 H. Fr. 11B (283.0 mg) was separated by ODS column chromatography (φ 20 × 210 mm; H_2_O-MeOH = 10:90–0:1) to give the fractions 4A to 4H. Fr. 12F (38.0 mg) was subjected to HPLC (Luna Phenyl-Hexyl; MeOH-H_2_O = 95:5, flow rate 2.0 mL/min) to afford compound **12** (2.2 mg, t_*R*_ 21.6 min) and compound **11** (19.2 mg, t_*R*_ 26.6 min).

*Compound* (**12**): Yellow solid; UV (MeOH) λ_max_ nm (logε): 364.5 (3.9), 306.5 (4.5), 278.5 (4.4); ^1^H and ^13^C NMR data, see Table 1; HRESIMS (negative) *m/z* 405.1327 [M-H]^−^ (calcd for C_24_H_21_O_6_, Δ −1.1 mmu); IR (ATR) v_max_: 3371, 2924, 1607, 1464, 1292, 1170 cm^−1^.

**Table 1:** ^1^H and ^13^C NMR Spectroscopic Data of **12** in CDCl_3_.

position	δ_C_ (100 MHz)	δ_H_ (600 MHz, *J* in Hz)
1	156.41^a^	
2	113.7	
3	159.2	
4	89.4	6.87 (1H, s)
4a	153.6	
5	101.6	6.85 (1H, s)
6	154.9	
7	142.5	
8	137.1	
8a	111.7	
9	183.5	
9a	104.5	
10a	156.38^a^	
1'	100.4	6.80 (1H, s)
2'	156.15^a^	
3'	132.3	
4'	113.1	5.72 and 5.16 (each 1H, s)
5'	19.2	2.10 (3H, s)
1′′	26.6	4.09 (2H, d, 5.8)
2′′	123.0	5.28 (1H, m)
3′′	132.3	
4′′	25.8	1.83 (3H, s)
5′′	18.2	1.69 (3H, s)
1-OH		14.10 (1H, s)
7-OCH_3_	62.1	3.79 (3H, s)

^a^These may be exchanged.

### Notch signaling transcriptional activity assay

In a 96-well white plate, Cells (LS174T Notch cells; 2 × 10^4^ cells per well) were cultured at 37 °C for 12 h. To express Notch1(1704–2531) protein in the cells, doxycycline (50 ng/mL) was added to each well, and cells were cultured at 37 °C for 12 h. Medium was changed to doxycycline-free medium containing each sample, then cells were incubated with at 37 °C for 12 h. A microplate luminometer (Thermo Fisher Scientific Inc., Waltham, USA) and the Bright-Glo™ Luciferase Assay System (Promega Co., Madison, USA) were used for measurement of Luciferase activity. Fluorometric Microculture Cytotoxicity Assay (FMCA) was used for measurement of cell viability of the sample-treated cells. After incubation of assay cells (2×10^4^ cells per well) in a 96-well black plate at 37 °C for 24 h, samples addition was performed at the same time as the luciferase assay. The assay cells were cultures at 37 °C for 12 h. A fluorescence plate reader (Thermo Fisher Scientific Inc., Waltham, USA) was used for determination of cell viability by FMCA.

### Cell culture

HPB-ALL cells were purchased from RIKEN BRC. Assay cells (LS174T Notch cells) and HEK293 cells were incubated in Dulbecco’s modified Eagle medium (DMEM) (Wako, Osaka, Japan) with 10% fetal bovine serum (FBS; BioWest, Nuaillé, France; Sigma-Aldrich Co. LLC., St. Louis, USA), Streptomycin (100 μg/mL) and Penicillin (100 unit/mL) (Sigma-Aldrich Co. LLC., St. Louis, USA). HPB-ALL cells were incubated in RPMI-1640 (Wako, Osaka, Japan) with 10% FBS, Streptomycin (100 μg/mL) and Penicillin (100 unit/mL). Cultures were maintained in a incubator at 37 °C in 5% CO_2_ and 95% air.

### Cytotoxicity test

HEK293 cells (1 × 10^4^ cells per well) were incubated in 96-well black plates in 100 μL of DMEM medium containing 10% FBS, Streptomycin (100 μg/mL) and Penicillin (100 unit/mL) at 37 °C for 24 h. The medium was exchanged to fresh medium with different concentrations of compound and cells were incubated for 72 h. Fluorometric microculture assay (FMCA) was used for determination of cell proliferation using a fluorescence plate reader (Thermo Fisher Scientific Inc., Waltham, USA).

HPB-ALL cells (1 × 10^4^ cells per well) were incubated in 96-well black plates in 100 μL of RPMI-1640 medium with 10% FBS, Streptomycin (100 μg/mL) and Penicillin (100 unit/mL). Media also contained different concentrations of compound. Cells were incubated at 37 °C for 72 h. After incubation, AlamarBlue (00–025, DAL1025; Thermo Fisher Scientific Inc., Waltham, USA) was added 10 μL per well. After incubation for 4 h, using a fluorescence plate reader (Thermo Fisher Scientific Inc., Waltham, USA), fluorescence was measured and cell viability was determined.

### Western blotting analysis

Cells (LS174T Notch cells; 1 × 10^6^ cells per dish) were incubated in a 6 cm dish for 12 h at 37 °C in 5 mL of DMEM medium with 10% FBS, Streptomycin (100 μg/mL) and Penicillin (100 unit/mL). Before another 12 h incubation, 50 ng/mL of doxycycline was added into each dish. Then, medium was changed to fresh medium with each compound. After 12 h incubation, cells were washed with PBS (500 μL) twice and then cells were collected by scraping. Using lysis buffer (20 mM Tris-HCl pH 7.4, 150 mM NaCl, 0.5% sodium deoxycholate, 10 mM EDTA, 1 mM sodium orthovanadate, and 0.1 mM NaF) containing a 1% proteasome inhibiotor cocktail (Nacalai Tesque, Tokyo, Japan), protein lysate was prepared. Protein lysates were separated by a 7.5%, 10%, 12.5% and 15% SDS-PAGE electrophoresis. Proteins were then transferred to a PVDF membrane (Bio-Rad Laboratories, Inc. Hercules, USA) electrophoretically. The membrane was incubated for 1 h with TBST (10 mM Tris-HCl pH 7.4, 100 mM NaCl and 0.1% Tween 20) containing 5% skimmed milk. Then, the membrane was mixed at 4 °C for 12 h with primary antibodies anti-β-actin (A2228; dilution 1:4000, Sigma-Aldrich, Co. LLC., St. Louis, USA), anti-cleaved Notch1 (Val1744) (2421, 4147; dilution 1:1000, Cell Signaling Technology, Inc., Danvers, USA), anti-FLAG (F7425; dilution 1:1000, Sigma-Aldrich, Co. LLC., St. Louis, USA), anti-HES1 (sc-13844; dilution 1:1000, Santa Cruz Biotechnology, Inc., Dallas, USA), anti-HES5 (ab25374; dilution 1:250, ab194111; dilution 1:1000, Abcam plc., Cambridge, UK), anti-aph1a (ab62821; dilution 1:1000, Abcam plc., Cambridge, UK), anti-nicastrin (5665; dilution 1:1000, Cell Signaling Technology, Inc., Danvers, USA), anti-presenilin1 (5643; dilution 1:1000, Cell Signaling Technology, Inc., Danvers, USA) or anti-pen2 (8598; dilution 1:1000, Cell Signaling Technology, Inc., Danvers, USA). Then, the TBST wash of membrane was performed and membrane was mixed at room temperature for 1 h with secondary antibodies either anti-rabbit IgG (111-035-144; dilution 1:4000, Jackson Immuno Research Inc., West Grove, USA), anti-rabbit IgG (7074; dilution 1:4000, Cell Signaling Technology, Inc., Danvers, USA), anti-goat IgG (705-035-003; dilution 1:10000, Jackson Immuno Research Inc., West Grove, USA) or anti-mouse IgG (NA931; dilution 1:4000, GE Healthcare Japan, Tokyo, Japan). After TBST was of membrane, detection of immunocomplexed bands were performed by an ECL Advance Western (RPN2135; GE Healthcare Japan, Tokyo, Japan), ECL Select Western (RPN2235; GE Healthcare Japan, Tokyo, Japan) or an Immobilon Western (WBKLS0100; Merck Millipore Co., Darmstadt, Germany) detection system.

HPB-ALL cells were incubated in a 6 cm dish in 5 mL of RPMI-1640 medium with 10% FBS, Streptomycin (100 μg/mL) and Penicillin (100 unit/mL) at 5 × 10^5^ cells per dish (1 × 10^6^ cells per dish, when treatment with MG132). Media also contained different concentrations of compound. Cells were incubated at 37 °C for 72 h (12 h, when treatment with MG132). After incubation, cells were centrifuged and collected, and then washed with 500 μL of PBS twice. To prepare cell lysate and confirm protein expression levels, the same method as described above was used.
